# Maternal health research outputs and gaps in Latin America: reflections from the mapping study

**DOI:** 10.1186/s12992-017-0300-2

**Published:** 2017-09-18

**Authors:** Emily Vargas-Riaño, Víctor Becerril-Montekio, Francisco Becerra-Posada, Mario Tristán

**Affiliations:** 1 CEO., EVidence Knowledge Brokering in Public Health SAS, Bogota, Colombia; 2 National Institute of Public Health (Instituto Nacional de Salud Pública)/ Centre for Health Systems Research, Segunda Privada Colorines 9, Colonia Santa María Ahuactatitlán, Cuernavaca, Morelos CP 62100 México; 3 Pan American Health Organization, Washington, D.C., USA; 4 International Health Central American Institute Foundation (IHCAI Foundation), San Jose, Costa Rica

**Keywords:** Maternal health, Literature mapping, Latin America, Research funding, MDGs

## Abstract

As part of the MASCOT/WOTRO multinational team conducting the maternal health literature mapping, four Latin American researchers were particularly interested in analysing information specific to their region. The mapping started with 45,959 papers uploaded from MEDLINE, CINAHL, Embase, LILACAS, PopLINE, PsycINFO and Web of Knowledge. From these, 4175 full texts were reviewed and 2295 papers were subsequently included. Latin America experienced an average maternal mortality decline of 40% between 1990 and 2013. Nevertheless, the region’s performance was below the global average and short of the 75% reduction set in Millennium Development Goal 5 for 2015. The main outcomes show that research on maternal health in the countries where the most impoverished populations of the world are living is not always aligned with their compelling needs. From another perspective, the review made it possible to recognize that research funding as well as the amount of scientific literature produced concentrate on issues that are not necessarily among the main causes of maternal deaths. Even though research on maternal health in Latin America has grown from an average of 92.5 publications per year in 2000-2003 to 236.7 between 2008 and 2012, it’s not satisfactorily keeping pace with other regions. In conclusion, it is critical to effectively orient research funding and production to respond to the health needs of the population. At the same time, there is a need for innovative mechanisms to strengthen the production and uptake of scientific evidence that can properly inform public health decision making.

## Background – The mapping study and Latin America

A literature mapping study is generally useful to identify relevant articles in a particular area of interest. It differs from a regular systematic review in that rather than searching for the best answers in solving a particular problem, a mapping study aims to identify how the theme or the area of interest has been approached by research. Thus, mapping studies may even disregard methodological soundness as secondary [1]. Based on this methodology, the MASCOT/WOTRO maternal health literature mapping [2] reviewed publications stemming from 2000 to 2012 on maternal health issues in low and middle income countries (LMICS) [3].

This mapping study was carried out by a group of 15 reviewers from eight countries across all five continents. As has already been reported in detail by Chersich et al. [2], the mapping started with a total 45,959 papers uploaded from MEDLINE, CINAHL, Embase, LILACAS, PopLINE, PsycINFO and Web of Knowledge. All duplicates (10,881) were eliminated, leaving 35,078 titles and abstracts that were independently screened by two-member teams. Again, all papers not related to maternal health were excluded (16,094), as well as those aiming clinical interventions other than the tracer conditions defined for the mapping review. A total 4175 full texts were reviewed out of which 2295 were included in the final mapping.

In most countries of Latin America and the Caribbean (LAC), maternal health is still a concerning issue. Even with an average maternal mortality decline of 40% between 1990 and 2013, the region’s performance was below the global average and behind the 75% reduction set by Millennium Development Goal (MDG) 5 for 2015 [4]. Notwithstanding the dramatic increase in the proportion of deliveries attended by skilled health personnel (18% to 92% between 1990 and 2014), profound inequalities in access to and use of reproductive health services still persist across the region and within countries, maintaining the largest difference in skilled delivery rate (20%) between rural and urban coverage worldwide [4].

In this context, four researchers from three Latin American countries joined the multinational team conducting the MASCOT/WOTRO maternal health literature mapping. They naturally exhibited a particular interest in research published in the nations within their region and the conclusions they could draw from those results.

## Is research addressing health needs?

As the various articles stemming from this mapping endeavour highlight from different perspectives, research on maternal health in the countries where the most impoverished populations are living is not always aligned with their compelling needs. One of the articles on this issue clearly states: “Several mismatches were noted between research publications, and the burden and causes of maternal deaths” [2].

Additionally, the review revealed that in many countries the majority of research funding went to HIV research. This is true in terms of quantity and funding of scientific research. It is undeniable that these countries need evidence-based solutions and well-informed policies to address HIV, however for example in Brazil 51% of the articles in the review were dedicated to HIV and much fewer studies examined the health system and health promotion or important maternal mortality causes such as maternal haemorrhage.

Nevertheless, addressing HIV in LAC does not seem so urgent. In 2013, 2.8% of maternal deaths in the Caribbean and 1.2% in all LAC were HIV-related, while the figures were 3.8% in sub-Saharan Africa and 2.6% globally [5]. At the same time, the epidemiological transition experienced by most LAC countries saw chronic diseases surpass infectious conditions as the main causes of maternal deaths. For example, haemorrhage accounts for an estimated 25% of deaths, hypertension for 23% and indirect causes such as malaria, HIV/AIDS and cardiovascular diseases for 17% [6]. Even considering the important variations among countries in the region it is clear that most preventable deaths occur in the course of labour and delivery, signalling the need to improve the quality of obstetric care [4]. Concerning the main topics of the review, Argentina dedicated over a third of its maternal health research to haemorrhage (36%), while research in Mexico covered a broader range of topics. This brings into question whether it is better for a country to develop considerable research expertise on a few priority conditions or to address a broader range of diseases. Ideally it may even be possible for different countries in LAC to coordinate their approach, focusing on specific conditions and subsequently sharing their findings.

## Is financing defining research agendas?

Even though research on maternal health in Latin America has grown from an average of 92.5 publications in 2000-2003 to 236.7 between 2008 and 2012, it is not satisfactorily keeping pace with other regions. This can be related to the fact that a considerable proportion (27%) of maternal health research in the countries that publish the most (Brazil, 6.2% of all the studies in the review, Mexico, 1.7% and Argentina 1.0%) is locally financed by government agencies. Private foundations and multilateral agencies are also important funders of maternal health research in Latin America. Nevertheless, several factors such as the urgency of addressing major threats like HIV may guide international funding to focus on other regions of the world, mainly Africa. This research financing environment could also explain why maternal health research in most LAC is still limited.

During the last 10 years, a range of stakeholders in health research, particularly in maternal health research, has gained prominence in LAC. This new context has fostered strong collaborations between different actors from academia, health system decision makers, non-government organizations and the community, but more participatory processes must be created. In order to achieve this some Latin American countries are increasingly leading and investing in health research and thus strengthening their capacities. The desired collaboration among LAC countries is beginning to become tangible [7].

## Are quality criteria limiting research?

One last element affecting the region’s capacity to expand its publication capacities is the use of the Impact Factor as a criterion to judge the quality of journals and the papers within them. This long-debated issue [8] remains a major concern, as is the relative bias implicit in the need to publish in English considering that the majority of Latin American researchers speak either Spanish or Portuguese. Even though the mapping made particular efforts to include papers published in these two languages [9], only 37.5% of the Latin American studies identified in this mapping were published in journals with an Impact Factor (which was used as a proxy for the quality of research). This percentage is lower than that of the other regions.

## Conclusions

Several conclusions can be drawn from the Latin American scenario that may be generalized to maternal health research outputs and gaps in most LMICs. First, sound research needs to be supported by valid data, which, in turn, demands a strengthening of health information systems. Secondly, it is critical to effectively orient research funding and production to respond to the health needs of the population. And finally, ever more innovative mechanisms must be utilized to strengthen the production and use of scientific evidence to properly inform public health decision making. In the case of Latin America, this last issue acquires a particular significance. While researchers are increasingly complying to the need to publish in (English language) high impact factor journals, this might be creating a bigger gap between high quality research production and its uptake by decision makers. All these conclusions gain particular relevance as countries start working towards the Sustainable Development Goals for 2030, where the reduction of maternal mortality remains an unfinished issue.

## Abbreviations

LAC: Latin America and the Caribbean
LMICS: Low and middle income countries
